# Full Genome Sequence of a *Methanomassiliicoccales* Representative Enriched from Peat Soil

**DOI:** 10.1128/MRA.00443-21

**Published:** 2021-12-02

**Authors:** Micha Weil, Katharina J. Hoff, Walter Meißner, Fabian Schäfer, Andrea Söllinger, Haitao Wang, Lisa Hagenau, Andreas W. Kuss, Tim Urich

**Affiliations:** a Institute of Microbiology, University of Greifswald, Greifswald, Germany; b Institute for Mathematics and Computer Science, University of Greifswald, Greifswald, Germany; c Human Molecular Genetics Group, Department of Functional Genomics, Interfaculty Institute for Genetics and Functional Genomics, University Medicine Greifswald, Greifswald, Germany; d Center for Functional Genomics of Microbes, University of Greifswald, Greifswald, Germany; Indiana University, Bloomington

## Abstract

The full genome of a *Methanomassiliicoccales* strain, U3.2.1, was obtained from enrichment cultures of percolation fen peat soil under methanogenic conditions, with methanol and hydrogen as the electron acceptor and donor, respectively. Metagenomic assembly of combined long-read and short-read sequences resulted in a 1.51-Mbp circular genome.

## ANNOUNCEMENT

Methane from methanogenic archaea in wetland peat soils contributes 33% to the global biological methane emissions (1). The methanogenic order *Methanomassiliicoccales* (2, 3), which was discovered in 2012, was detected in wetland soils worldwide, sometimes representing a substantial fraction of the methanogenic community (4–6). To date, only a single isolate has been validly described, Methanomassiliicoccus luminyensis 10B, which was isolated from human feces (2). A draft genome of M. luminyensis and a few complete genomes of *Methanomassiliicoccales* enrichment cultures, obtained mainly from gastrointestinal tracts of animals and humans but also from the environment, have been described since then (4, 7–12). However, no highly enriched *Methanomassiliicoccales* cultures or complete genomes from peat soil have been obtained thus far. Here, we present the complete circular genome of a *Methanomassiliicoccales* member enriched from peat soil from a percolation fen, strain U3.2.1.

The peat used for enrichment was sampled in a percolation fen in northeastern Germany (54.10N, 12.74E), at a depth of 25 cm (13). Modified MpT1 medium, with methanol as the electron acceptor under an anaerobic H_2_/CO_2_ atmosphere, was used for the enrichment of *Methanomassiliicoccales* as described previously. Enrichment success was monitored with quantitative PCR (4). DNA was extracted with a phenol/chloroform-based DNA extraction protocol (14). Gene quantification was done by quantitative PCR, using the primer pairs AS1/AS2 and EuFor/EuRev for *Methanomassiliicoccales* and bacteria, respectively (15, 16). After several rounds of culture incubations for 2 weeks and consecutive transfers, 16S rRNA genes of *Methanomassiliicoccales* accounted for 60% of the total prokaryotic 16S rRNA genes. Paired-end-read Illumina MiSeq v4 sequencing was performed by LGC Genomics (Berlin, Germany) using the primer pair 515YF/B806R for prokaryotes (17). The raw sequence reads were processed by demultiplexing and removing barcodes, adapters, and primers using the Illumina bcl2fastq software. The data were then quality filtered with the DADA2 v1.8.0 pipeline (18) in R v3.5. The data revealed the presence of a single 16S rRNA gene sequence of *Methanomassiliicoccales* in several of the enrichment cultures.

Without DNA shearing and size selection, long-read sequencing libraries were prepared from enrichment culture U.3.2.1 using the ligation sequencing kit (SQK-LSK109; Oxford Nanopore Technologies) and then were sequenced on a MinION Mk1B sequencer (Oxford Nanopore Technologies) using the kit flow cell (R9.4.1), producing 3.43 million reads (12.2 Gb). Base calling for MinION reads was performed with Guppy v2.3.7 with the flip-flop model. For short-read sequencing, libraries were prepared from the same DNA extract with the NEBNext Ultra II DNA library preparation kit (New England Biolabs) and sequenced on a MiSeq platform with a MiSeq reagent microkit v2 (Illumina). MinION reads were quality trimmed and filtered with NanoFilt v2.3.0 (19) with parameters -l 2000 -q 7. This resulted in 3,299,097 reads, with an *N*_50_ value of 1,099,330 nucleotides. In the following steps, default parameters were used. An initial metagenome assembly was generated with MetaSPAdes v3.11.0 (20). MinION reads were mapped against the assembly using minimap2 v2.17-r943-dirty (21), and Illumina reads were mapped to the assembly with BWA v0.7.17-r1188 (22). Contigs of the initial assembly were searched for hits in the UniProt reference proteomes (23) (https://ftp.uniprot.org/pub/databases/uniprot/current_release/knowledgebase/complete/uniprot_sprot.fasta.gz [accessed 25 October 2018]) with DIAMOND v0.9.2 (24). BlobTools v1.1.1 (25) was used to find contigs that are likely of archaeal origin. MinION reads that mapped to these contigs were selected with SAMtools v1.7 (26). The selected MinION reads together with Illumina reads were assembled with SPAdes v3.11.0 (27, 28). The resulting scaffolds were binned with Centrifuge v1.0.4 (29). One scaffold was classified as archaeal by Centrifuge. This scaffold was polished twice with RACON v1.3.3 (30) using MinION reads and twice with Pilon v1.23 (31) using Illumina reads. NCBI BLAST v2.9.0+ was used to search for sequence similarity in the two ends of the linear genome. This resulted in an overlap of 56 bp. The overlapping 56 bp were removed from the end of the genome sequence. This metagenome assembly workflow resulted in a high-quality circular genome of 1.51 Mbp.

The circular genome enabled a thorough analysis of the genomic potential using Rapid Annotation using Subsystems Technology (RAST) (32, 33). The genome had a GC content of 43.7%, with 1,535 coding gene sequences, 44 tRNA genes, and 4 rRNA genes (one 16S rRNA gene, one 23S rRNA gene, and two 5S rRNA genes). The closest relative, based on the 16S rRNA gene sequence, was “*Candidatus* Methanogranum caenicola,” with 97.7% sequence identity; the sequence identity to the 16S rRNA gene of Methanomassiliicoccus luminyensis 10B was 88.6%.

### Data availability.

The genome of *Methanomassiliicoccales* strain U3.2.1 is available in the NCBI database, with GenBank accession number CP076745.1. The whole-genome sequencing data are available under NCBI BioProject accession number PRJNA731838, with taxonomic identification number 1799672.

## References

[1] Lyu Z, Shao N, Akinyemi T, Whitman WB. 2018. Methanogenesis. Curr Biol 28:R727–R732. doi:10.1016/j.cub.2018.05.021. 29990451

[2] Dridi B, Fardeau ML, Ollivier B, Raoult D, Drancourt M. 2012. *Methanomassiliicoccus luminyensis* gen. nov., sp. nov., a methanogenic archaeon isolated from human faeces. Int J Syst Evol Microbiol 62:1902–1907. doi:10.1099/ijs.0.033712-0. 22859731

[3] Nkamga VD, Drancourt M. 2016. *Methanomassiliicoccales*. *In* Trujillo ME, Dedysh S, DeVos P, Hedlund B, Kämpfer P, Rainey FA, Whitman WB (ed), Bergey's manual of systematics of archaea and bacteria. John Wiley & Sons, Inc, Hoboken, NJ. doi:10.1002/9781118960608.gbm01365.

[4] Söllinger A, Schwab C, Weinmaier T, Loy A, Tveit AT, Schleper C, Urich T. 2016. Phylogenetic and genomic analysis of *Methanomassiliicoccales* in wetlands and animal intestinal tracts reveals clade-specific habitat. FEMS Microbiol Ecol 92:fiv149. doi:10.1093/femsec/fiv149. 26613748

[5] Narrowe AB, Borton MA, Hoyt DW, Smith GJ, Daly RA, Angle JC, Eder EK, Wong AR, Wolfe RA, Pappas A, Bohrer G, Miller CS, Wrighton KC. 2019. Uncovering the diversity and activity of methylotrophic methanogens in freshwater wetland soils. mSystems 4:e00320-19. doi:10.1128/mSystems.00320-19. 31796563 PMC6890927

[6] Yang S, Liebner S, Winkel M, Alawi M, Horn F, Dörfer C, Ollivier J, He J-s, Jin H, Kühn P, Schloter M, Scholten T, Wagner D. 2017. In-depth analysis of core methanogenic communities from high elevation permafrost-affected wetlands. Soil Biol Biochem 111:66–77. doi:10.1016/j.soilbio.2017.03.007.

[7] Gorlas A, Robert C, Gimenez G, Drancourt M, Raoult D. 2012. Complete genome sequence of *Methanomassiliicoccus luminyensis*, the largest genome of a human-associated *Archaea* species. J Bacteriol 194:4745. doi:10.1128/JB.00956-12. 22887657 PMC3415480

[8] Noel SJ, Højberg O, Urich T, Poulsen M. 2016. Draft genome sequence of “Candidatus Methanomethylophilus” sp. 1R26, enriched from bovine rumen, a methanogenic archaeon belonging to the Methanomassiliicoccales order. Genome Announc 4:e01734-15. doi:10.1128/genomeA.01734-15. 26893425 PMC4759072

[9] Borrel G, Harris HMB, Tottey W, Mihajlovski A, Parisot N, Peyretaillade E, Peyret P, Gribaldo S, O’Toole PW, Brugère J-F. 2012. Genome sequence of “*Candidatus* Methanomethylophilus alvus” Mx1201, a methanogenic archaeon from the human gut belonging to a seventh order of methanogens. J Bacteriol 194:6944–6945. doi:10.1128/JB.01867-12. 23209209 PMC3510639

[10] Cozannet M, Borrel G, Roussel E, Moalic Y, Allioux M, Sanvoisin A, Toffin L, Alain K. 2020. New insights into the ecology and physiology of *Methanomassiliicoccales* from terrestrial and aquatic environments. Microorganisms 9:30. doi:10.3390/microorganisms9010030. PMC7824343 33374130

[11] Li Y, Leahy SC, Jeyanathan J, Henderson G, Cox F, Altermann E, Kelly WJ, Lambie SC, Janssen PH, Rakonjac J, Attwood GT. 2016. The complete genome sequence of the methanogenic archaeon ISO4-H5 provides insights into the methylotrophic lifestyle of a ruminal representative of the *Methanomassiliicoccales*. Stand Genomic Sci 11:59. doi:10.1186/s40793-016-0183-5. 27602181 PMC5011839

[12] Kelly WJ, Li D, Lambie SC, Jeyanathan J, Cox F, Li Y, Attwood GT, Altermann E, Leahy SC. 2016. Complete genome sequence of methanogenic archaeon ISO4-G1, a member of the *Methanomassiliicoccales*, isolated from a sheep rumen. Genome Announc 4:e00221-16. doi:10.1128/genomeA.00221-16. 27056226 PMC4824259

[13] Weil M, Wang H, Bengtsson M, Köhn D, Günther A, Jurasinski G, Couwenberg J, Negassa W, Zak D, Urich T. 2020. Long-term rewetting of three formerly drained peatlands drives congruent compositional changes in pro- and eukaryotic soil microbiomes through environmental filtering. Microorganisms 8:550. doi:10.3390/microorganisms8040550. PMC7232337 32290343

[14] Leininger S, Urich T, Schloter M, Schwark L, Qi J, Nicol GW, Prosser JI, Schuster SC, Schleper C. 2006. Archaea predominate among ammonia-oxidizing prokaryotes in soils. Nature 442:806–809. doi:10.1038/nature04983. 16915287

[15] Mihajlovski A, Doré J, Levenez F, Alric M, Brugère JF. 2010. Molecular evaluation of the human gut methanogenic archaeal microbiota reveals an age-associated increase of the diversity. Environ Microbiol Rep 2:272–280. doi:10.1111/j.1758-2229.2009.00116.x. 23766078

[16] Lee DH, Zo YG, Kim SJ. 1996. Nonradioactive method to study genetic profiles of natural bacterial communities by PCR-single-strand-conformation polymorphism. Appl Environ Microbiol 62:3112–3120. doi:10.1128/aem.62.9.3112-3120.1996. 8795197 PMC168103

[17] Walters W, Hyde ER, Berg-Lyons D, Ackermann G, Humphrey G, Parada A, Gilbert JA, Jansson JK, Caporaso JG, Fuhrman JA, Apprill A, Knight R. 2016. Improved bacterial 16S rRNA gene (V4 and V4-V5) and fungal internal transcribed spacer marker gene primers for microbial community surveys. mSystems 1:e0009-15. doi:10.1128/mSystems.00009-15. PMC5069754 27822518

[18] Callahan BJ, McMurdie PJ, Rosen MJ, Han AW, Johnson AJA, Holmes SP. 2016. DADA2: high-resolution sample inference from Illumina amplicon data. Nat Methods 13:581–583. doi:10.1038/nmeth.3869. 27214047 PMC4927377

[19] De Coster W, D’Hert S, Schultz DT, Cruts M, Van Broeckhoven C. 2018. NanoPack: visualizing and processing long-read sequencing data. Bioinformatics 34:2666–2669. doi:10.1093/bioinformatics/bty149. 29547981 PMC6061794

[20] Nurk S, Meleshko D, Korobeynikov A, Pevzner PA. 2017. metaSPAdes: a new versatile metagenomics assembler. Genome Res 27:824–834. doi:10.1101/gr.213959.116. 28298430 PMC5411777

[21] Li H. 2018. Minimap2: pairwise alignment for nucleotide sequences. Bioinformatics 34:3094–3100. doi:10.1093/bioinformatics/bty191. 29750242 PMC6137996

[22] Li H, Durbin R. 2009. Fast and accurate short read alignment with Burrows-Wheeler transform. Bioinformatics 25:1754–1760. doi:10.1093/bioinformatics/btp324. 19451168 PMC2705234

[23] UniProt Consortium. 2015. UniProt: a hub for protein information. Nucleic Acids Res 43:D204–D212. doi:10.1093/nar/gku989. 25348405 PMC4384041

[24] Buchfink B, Xie C, Huson DH. 2015. Fast and sensitive protein alignment using DIAMOND. Nat Methods 12:59–60. doi:10.1038/nmeth.3176. 25402007

[25] Laetsch DR, Blaxter ML. 2017. BlobTools: interrogation of genome assemblies. F1000Res 6:1287. doi:10.12688/f1000research.12232.1.

[26] Li H, Handsaker B, Wysoker A, Fennell T, Ruan J, Homer N, Marth G, Abecasis G, Durbin R, 1000 Genome Project Data Processing Subgroup. 2009. The Sequence Alignment/Map format and SAMtools. Bioinformatics 25:2078–2079. doi:10.1093/bioinformatics/btp352. 19505943 PMC2723002

[27] Bankevich A, Nurk S, Antipov D, Gurevich AA, Dvorkin M, Kulikov AS, Lesin VM, Nikolenko SI, Pham S, Prjibelski AD, Pyshkin AV, Sirotkin AV, Vyahhi N, Tesler G, Alekseyev MA, Pevzner PA. 2012. SPAdes: a new genome assembly algorithm and its applications to single-cell sequencing. J Comput Biol 19:455–477. doi:10.1089/cmb.2012.0021. 22506599 PMC3342519

[28] Antipov D, Korobeynikov A, McLean JS, Pevzner PA. 2016. HybridSPAdes: an algorithm for hybrid assembly of short and long reads. Bioinformatics 32:1009–1015. doi:10.1093/bioinformatics/btv688. 26589280 PMC4907386

[29] Kim D, Song L, Breitwieser FP, Salzberg SL. 2016. Centrifuge: rapid and sensitive classification of metagenomic sequences. Genome Res 26:1721–1729. doi:10.1101/gr.210641.116. 27852649 PMC5131823

[30] Vaser R, Sović I, Nagarajan N, Šikić M. 2017. Fast and accurate de novo genome assembly from long uncorrected reads. Genome Res 27:737–746. doi:10.1101/gr.214270.116. 28100585 PMC5411768

[31] Walker BJ, Abeel T, Shea T, Priest M, Abouelliel A, Sakthikumar S, Cuomo CA, Zeng Q, Wortman J, Young SK, Earl AM. 2014. Pilon: an integrated tool for comprehensive microbial variant detection and genome assembly improvement. PLoS One 9:e112963. doi:10.1371/journal.pone.0112963. 25409509 PMC4237348

[32] Aziz RK, Bartels D, Best A, DeJongh M, Disz T, Edwards RA, Formsma K, Gerdes S, Glass EM, Kubal M, Meyer F, Olsen GJ, Olson R, Osterman AL, Overbeek RA, McNeil LK, Paarmann D, Paczian T, Parrello B, Pusch GD, Reich C, Stevens R, Vassieva O, Vonstein V, Wilke A, Zagnitko O. 2008. The RAST Server: Rapid Annotations using Subsystems Technology. BMC Genomics 9:75. doi:10.1186/1471-2164-9-75. 18261238 PMC2265698

[33] Overbeek R, Olson R, Pusch GD, Olsen GJ, Davis JJ, Disz T, Edwards RA, Gerdes S, Parrello B, Shukla M, Vonstein V, Wattam AR, Xia F, Stevens R. 2014. The SEED and the Rapid Annotation of microbial genomes using Subsystems Technology (RAST). Nucleic Acids Res 42:D206–D214. doi:10.1093/nar/gkt1226. 24293654 PMC3965101

